# Structural studies of the *Enterococcus faecalis *SufU [Fe-S] cluster protein

**DOI:** 10.1186/1471-2091-10-3

**Published:** 2009-02-02

**Authors:** Gustavo P Riboldi, Hugo Verli, Jeverson Frazzon

**Affiliations:** 1 Programa de Pós-Graduação em Biologia Celular e Molecular, Centro de Biotecnologia, Universidade Federal do Rio Grande do Sul (UFRGS), Porto Alegre, Brazil; 2 Faculdade de Farmácia, UFRGS, Porto Alegre, Brazil; 3 Instituto de Ciências e Tecnologia de Alimentos, UFRGS, Porto Alegre, Brazil

## Abstract

**Background:**

Iron-sulfur clusters are ubiquitous and evolutionarily ancient inorganic prosthetic groups, the biosynthesis of which depends on complex protein machineries. Three distinct assembly systems involved in the maturation of cellular Fe-S proteins have been determined, designated the NIF, ISC and SUF systems. Although well described in several organisms, these machineries are poorly understood in Gram-positive bacteria. Within the *Firmicutes *phylum, the *Enterococcus *spp. genus have recently assumed importance in clinical microbiology being considered as emerging pathogens for humans, wherein *Enterococcus faecalis *represents the major species associated with nosocomial infections. The aim of this study was to carry out a phylogenetic analysis in *Enterococcus faecalis V583 *and a structural and conformational characterisation of it SufU protein.

**Results:**

BLAST searches of the *Enterococcus *genome revealed a series of genes with sequence similarity to the *Escherichia coli *SUF machinery of [Fe-S] cluster biosynthesis, namely *sufB, sufC, sufD *and *SufS*. In addition, the *E. coli *IscU ortholog SufU was found to be the scaffold protein of *Enterococcus spp.*, containing all features considered essential for its biological activity, including conserved amino acid residues involved in substrate and/or co-factor binding (Cys^50,76,138 ^and Asp^52^) and, phylogenetic analyses showed a close relationship with orthologues from other Gram-positive bacteria. Molecular dynamics for structural determinations and molecular modeling using *E. faecalis *SufU primary sequence protein over the PDB:1su0 crystallographic model from *Streptococcus pyogenes *were carried out with a subsequent 50 ns molecular dynamic trajectory. This presented a stable model, showing secondary structure modifications near the active site and conserved cysteine residues. Molecular modeling using *Haemophilus influenzae *IscU primary sequence over the PDB:1su0 crystal followed by a MD trajectory was performed to analyse differences in the C-terminus region of Gram-positive SufU and Gram-negative orthologous proteins, in which several modifications in secondary structure were observed.

**Conclusion:**

The data describe the identification of the SUF machinery for [Fe-S] cluster biosynthesis present in the *Firmicutes *genome, showing conserved *sufB, sufC, sufD *and *sufS *genes and the presence of the *sufU *gene coding for scaffold protein, instead of *sufA*; neither *sufE *nor *sufR *are present. Primary sequences and structural analysis of the SufU protein demonstrated its structural-like pattern to the scaffold protein IscU nearby on the ISC machinery. *E. faecalis *SufU molecular modeling showed high flexibility over the active site regions, and demonstrated the existence of a specific region in *Firmicutes *denoting the Gram positive region (GPR), suggested as a possible candidate for interaction with other factors and/or regulators.

## Background

Iron-sulfur [Fe-S] clusters are simple inorganic prosthetic groups that are widely distributed in nature and play essential roles in diverse biological processes such as electron transfer, redox and nonredox catalysis, gene regulation and as sensors within all living organisms [1-4]. The biosynthetic process by which defined proportions of iron and sulfur atoms are mobilised and combined to generate the various iron-sulfur prosthetic groups within polypeptide chains has been a matter of intensive research during the last 10 years. The cluster components iron (ferrous or ferric forms) and sulphide ions are unavailable in cytosolic solutions due to their toxicity, making it unlikely that [Fe-S] clusters are synthesised by apoproteins from free cytosolic iron and sulphide elements, even though this is a rather efficient process *in vitro *[5]. Therefore, the functions of the [Fe-S] cluster assembly machineries include the mobilisation of Fe^2+/3+ ^and S^2- ^elements from their storage sources, their association into an [Fe-S] bound form and its transport and transfer to the final molecular destinations. Despite the apparent diversity in the overall structure, reactivity, electronic properties and molecular environments of [Fe-S] clusters, previous data have demonstrated that [4Fe-4S] clusters, as well as clusters of even higher nuclearity, are chemically assembled by the reductive coupling of [2Fe-2S] units [6-9].

The machinery of [Fe-S] biogenesis is represented by at least three distinct, yet structurally and functionally related systems, designated NIF, ISC, and SUF. The NIF system, in addition to performing specialised functions in nitrogen fixation and subsequent maturation of the nitrogenase enzyme, is formed by structural and regulatory genes represented by around 20 genes [10,11]. The ISC system for iron-sulfur cluster assembly probably represents the housekeeping system for [Fe-S] protein maturation in most living cells and is comprised of *iscRSUA-hscBA-fdx *genes [12]. The SUF system performs its role in sulfur assimilation, is comprised of *sufABCDES *genes and occurs in numerous bacteria, in archaea, and in plant chloroplasts [13,14].

All three operons contain genes that encode proteins with similar biochemical activity [15]. Accordingly, NifU/IscU/SufU scaffold proteins have characteristic primary sequences, the NifU protein possessing the complete form with 312 amino acid residues and 9 conserved cysteine residues. NifU contains three domains denoted the N-terminal domain (3 conserved cysteines), the central domain (4 conserved cysteines) and the C-terminal domain (2 conserved cysteines). In addition, the N-terminal domain of NifU corresponds to IscU (120 amino acids) and SufU (136 amino acids) proteins and appears to be involved in the formation and delivery of a transient [Fe-S] cluster [16]. A fourth conserved amino acid represented by an aspartic residue located immediately after the first conserved cysteine residue exhibits a critical role in the [Fe-S] cluster delivery: its substitution substantially stabilises the cluster, obstructing the donation of the cluster to the target protein [17].

In addition to the NifU/IscU/SufU scaffold proteins, the cysteine desulfurase NifS/IscS/SufS proteins appear to play essential roles in [Fe-S] cluster formation [18]. Such proteins possess an active cysteine residue (Cys^365^), involved in desulfuration of the pyridoxal-5'-phosphate (PLP) cofactor and thus in donating the sulfur element to the scaffold protein. Each NIF, ISC and SUF system has particular genes, such as the heat shock determinants (*hscAB*), the alternative scaffold protein IscA in the ISC machinery and the cysteine desulfurase enhancer SufE in the SUF system.

The SUF system has been linked to virulence in several microorganisms, as in *Mycobacterium tuberculosis*, whose *pps1 *gene codes for an orthologous SufB protein. It has been established that Pps1 is a central element of the SUF system, playing an essential role in *M. tuberculosis *survival through its involvement in the bacterial resistance to iron limitation and oxidative stress [19]. In *Erwinia chrysanthemi*, which causes soft-rot disease in a great variety of plants, iron acquisition and resistance to oxidative stress greatly contribute to its virulence, as *sufA *and *sufC *mutants exhibit reduced ability to cause maceration of leaves, while a functional *sufC *gene is required for the bacteria to cause systemic invasion [20]. Altogether, these data point to the use of such a system as a possible molecular target for the development of bioactive compounds able to modulate several biological processes. This appears to be particularly important in Gram-positive bacteria, considering the high virulence associated with bacteria belonging to the *Firmicutes *phylum where only the SUF system is present. A particularly important genus belong from this phylum may be found in *Enterococcus *spp., which comprises more than 20 species. Clinically, enterococci have been identified as second agents responsible by nosocomial infections wherein *Enterococcus faecalis *species accounting for 80–90% of clinical isolates. [21].

Several crystal and NMR structures of proteins involved in [Fe-S] cluster formation machinery have been described for numerous organisms, such as IscA [22], SufA [23], SufE [24], IscU [25] and SufU [26]. However, no structural information has been available related to proteins present in microorganisms belonging to the *Firmicutes *phyla. In this context, we report here the *in silico *molecular analysis of [Fe-S] cluster biosynthetic machinery in the *Firmicutes E. faecalis*, as well as molecular modeling data of the scaffold proteins SufU from *E. faecalis *and IscU from *S. pyogenes*. Overall, the analysis identified putative structural differences associated with Gram-negative (IscU protein) and Gram-positive (SufU proteins) bacteria, supporting structural and conformational differences in the participation of distinct machinery, which may ultimately be related to original and selective therapeutic strategies.

## Results and discussion

### The [Fe-S] cluster assembly machinery in *E. faecalis *and its conservative pattern in Gram-positive bacteria

**Figure 1:**
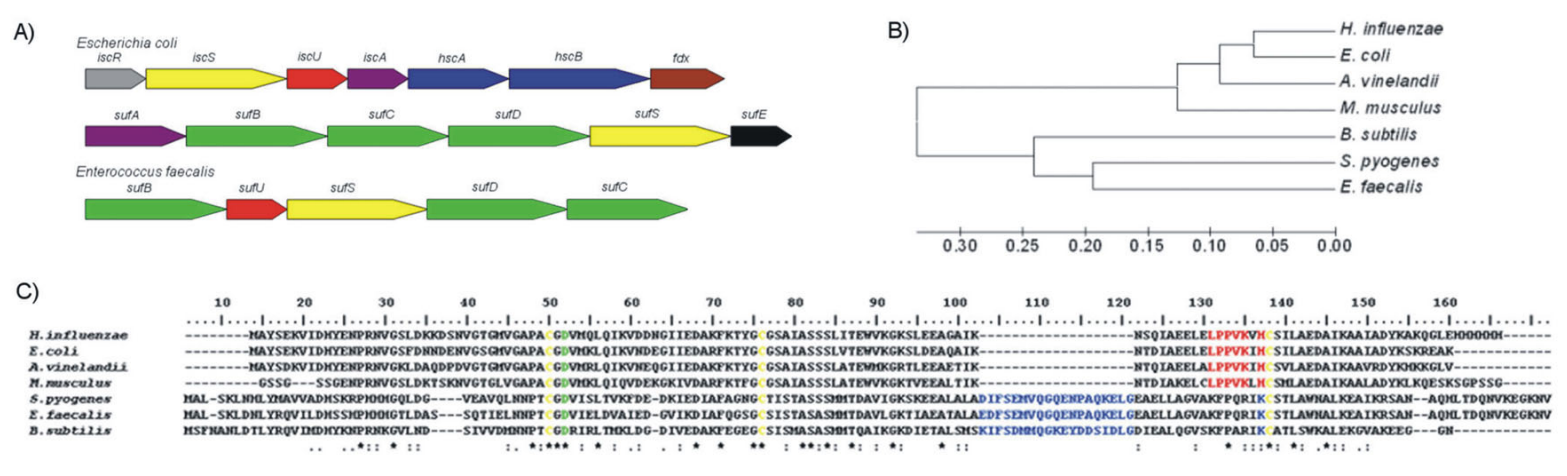
**The biosynthetic machinery for [Fe-S] cluster formation in Gram-positive bacteria**. (A) Comparison of the genetic organization of genes involved in the [Fe-S] cluster assembly. Genes having homologous sequences or similar functions between the two systems are color-coded: *E. coli *ISC and SUF machineries and conserved ORFs coding for putative SUF machinery in Gram-positive bacteria. (B) Neighbour-Joining phylogenetic analysis of protein sequences. (C) Comparison of sequences from members of the NifU/IscU/SufU orthologues. Cysteines are presented as yellow, aspartate as green, LPPVK of IscU in red, and the characteristic Gram-positive insertion in blue.

Bioinformatics analysis of the *E. faecalis *V583 genome [27] demonstrated the presence of a gene locus coding for putative SUF proteins (Fig. 1A). ORFs possibly related to [Fe-S] cluster biosynthetic machinery found in *E. faecalis *are denoted as: EF2390, harbouring 37% identity with SufB from *E. coli*; EF2391, encoding the putative scaffold NifU-like protein, homologous to IscU, the N-terminal module of NifU, which will be referred to in this work as SufU (according to Johnson *et al.*, 2005) [3]; EF2392, coding the cysteine desulfurase SufS protein, involved in donation of the sulfur molecule to the [Fe-S] cluster formation; EF3293 and EF2394 encoding SufD and SufC orthologue proteins, which presented 22% and 52% identity with the *E. coli *proteins, respectively. Once the phylogenetic analysis enabled the verification of high conservation and similarity for the *suf*BUSDC operon within *Firmicutes *phylum and, given *E. faecalis *clinical relevance, this work was performed having Gram-positive *E. faecalis *V583 strain as our model of study.

NifU/IscU/SufU is considered key proteins of their respective [Fe-S] cluster assembly machineries in bacteria. The IscU and SufU scaffold proteins show high homology with the N-terminal part of NifU, preserving the three conserved cysteines (Cys^50,76,138^) and aspartic residues (Asp^52^) (Fig. 1B and 1C). These three cysteine residues are proposed to be involved in the coordination of [Fe-S] cluster assembly with one noncysteinyl ligation, and the conserved aspartic residue exhibits a critical role in the [Fe-S] cluster formation, as its substitution substantially stabilizes the cluster, obstructing the donation of the cluster to the target protein. Moreover, IscU contains the conserved amino acid annotation LPPVK, which is related to the interaction with the heat-shock like proteins HscA and HscB, present only in such machinery [28]. *E. faecalis *SufU does not contain the LPPVK annotation, but does have an insertion of 19 amino acid residues between the second and third conserved cysteine residues. The annotation of these 19 amino acids was found to correspond to a specific signature for Gram-positive SufU-type proteins and is thought to replace the annotation present in IscU, since it is localised in a related region. Furthermore, the same type of *suf *gene representations were identified in several other members of Gram-positive bacteria, such as microorganisms phylogenetically clustered within the *Streptococcus*, *Staphylococcus*, *Bacillus *and *Listeria *genera, important microorganisms related to pathological processes, along with *Lactococcus *and *Lactobacillus *industrially relevant microorganisms (additional file 1). In the bacterial phyla, the Gram-positive forms make up the phylum *Firmicutes*, which includes many well-known genera such as *Bacillus*, *Listeria*, *Staphylococcus*, *Streptococcus*, *Enterococcus*, and *Clostridium*. In fact, phylogenetic analyses of IscU and SufU protein sequences suggested that SufU and IscU proteins form well-defined clades (additional file 2).

Comparison between *suf *genes observed in *Proteobacteria *and *Firmicutes *demonstrated several differences. SufE, for example, is an important protein present in the *Proteobacteria suf *operon, but whose presence has not been demonstrated in Gram-positive genomes. This protein is able to drastically stimulate the cysteine desulfurase activity of SufS [29,30]. Another protein present in the *E. coli *operon and absent in Gram-positive sequences is SufA, whose function is to accept the sulfur transferred from the SufS-SufE complex for transient [Fe-S] cluster formation. Furthermore, cyanobacteria were found to contain the *sufR *gene, which encodes a protein that functions as a transcriptional repressor of the *suf *regulon [31], but it was not found in the *Firmicutes *genomes. On the other hand, Gram-positive genomes contained SufU as a scaffold protein, which is unusual in the *Proteobacteria suf *locus. Finally, it can be assumed that four genes are present in both *Proteobacteria *and *Firmicutes *genomes, i.e., *sufB, sufC, sufD *and *sufS*. Interestingly, published data have always linked the SUF machinery for [Fe-S] cluster formation with cellular stress conditions, such as the presence of reactive oxygen species (ROS), NO stress and iron starvation [20]. But at what point is the SUF machinery that is involved in maturation of constitutive proteins related to other mechanisms in such microorganisms, since it seems to be the only protein factory present in *E. faecalis *as well as in other *Firmicutes *genomes? This is an intriguing and open question in this field, which needs biochemical and structural data for better comprehension.

All data described until now have demonstrated an unusual pattern of gene presentation, especially given the odd SUF-scaffold protein. Thus, we focused subsequent steps of the study on further analysing SufU structure and folding, with the aim of identifying important regions and comparing them to the patterns of [Fe-S] cluster-scaffold proteins determined in *Proteobacteria*.

### The structure and molecular modeling of the *E. faecalis *SufU protein

**Figure 2:**
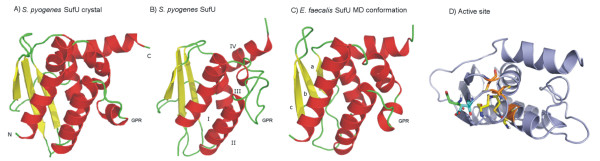
**Representation of crystallographic and MD conformations of SufU**. (A) Template *S. pyogenes *SufU crystal (PDB ID 1SU0); (B) SufU *S. pyogenes *conformation after 50 ns; (C) SufU *E. faecalis *model conformation after 50 ns. SufU structural characteristics are presented (α-helices I-IV and β-sheet a-c), as well as the characteristic Gram-positive region of 19 amino acids (GPR). (D) Active showing different conformations of active sites residues before and after MD trajectory: Cys residues are shaded in yellow and orange, and Asp in green and cyan blue for crystal and MD conformations, respectively.

*Firmicutes *SufU proteins exhibits a tertiary fold that is composed of four α-helices, I to IV, which comprise amino acids residues 8–19, 66–77, 84–97, and 105–141, respectively, and a three-stranded anti-parallel β-sheet with strands A to C comprising, respectively, residues 33–37, 42–49, and 57–63 (Fig. 2). Helices III and VI form a "coiled-coil" motif, and both helices are attached to one side of the β-sheet with helix III being oriented parallel to strand C. Helix IV is oriented approximately anti-parallel to helix III. The remaining helices I, II, and V surround helix III, with helix III buried in the protein's core [26]. The CATH protocol assigns SufU to the "a-b" "fold" class having a "two-layer sandwich" architecture, and as "SufE-like" in the class of a and b proteins in the SCOP classification [24].

In order to characterize the structure and conformation of the SufU key protein observed in *E. faecalis *comparative modeling was carried out [32] using the *S. pyogenes *(PDB:1SU0) [24]. SufU was employed as a template as due to its 43% of primary sequence similarity with the target. The quality of the so obtained model was checked through Procheck, PSIPRED and VERIFY3D. The *E. faecalis *SufU model maintained the tertiary fold as observed for *S. pyogenes*.

Although containing most of the genes related to the SUF system of *Proteobacteria*, Gram-positive bacteria also contained the scaffold protein with the active site conserved residues and tridimensional structure resembling IscU from the ISC system. Neither the primary composition nor the three-dimensional structure seen in the scaffold protein SufU matches SufA, the scaffold-function protein found in the *E. coli *SUF system, but it does match the *Proteobacteria *IscU structure [25]. The active site (Fig. 2D) contains all three conserved Cys and Asp residues that allow the formation of the transient [2Fe-2S] cluster for posterior delivery to target proteins, as verified in SufU. The union of two subunits of this protein to form a homodimer, with the reduction of two [2Fe-2S]^2+ ^to form a [4Fe-4S] cluster, is feasible [34]. Thus, SufU follows not only the IscU folding pattern but also has flexibility in the active site, which tends to be the region with the biggest structural alterations, and which enables the cluster formation, reduction, and monomer/homodimer alterations in the protein.

As already described, any sequence matching of the SufE primary structure was obtained in *E. faecalis *genome analysis. However, according to Liu *et al. *(2005) [26], although sharing < 10% sequence identity, the similarity of the three-dimensional structure suggests that IscU/SufU and SufE are homologous desulfurase enhancers. Thus, SufU could be the molecule responsible for scaffold and desulfurase enhancer actions in Gram-positive SUF machinery. In *Proteobacteria*, this function is exerted by two different molecules, namely SufA and SufE.

As reported [34,35], crystallographic structures may retain non-biological conformations as due to forces occurring in the crystal environment. By employing such structures in comparative modeling these conformational events associated with crystal packing may be transported to the model, potentially compromising the analysis of the obtained structures. One strategy capable to circumvent such effects, complementing the crystallographic information upon addition of solvent components and molecules flexibility, may be found in molecular dynamics (MD) simulations. Indeed, such strategy has already been employed with success to refine comparative models of proteins, allowing the observation of modifications on secondary structure elements of the interest protein towards the NMR observed structure [32,36,37]. So, the obtained model was further refined through MD simulations and, to trace possible crystal packing effects, the template *S. pyogenes *SufU was also simulated in the same conditions.

Molecular dynamics (MD) experiments with crystal data obtained from the *Firmicutes S. pyogenes *were performed in order to analyse the structural pattern of SufU under physiological conditions and remove possible crystal effects on the structure. MD results showed consistent changes in the secondary structure of *S. pyogenes *SufU after 50 ns of trajectory. Changes were verified both over the four α-helices, comprising residues 4–14, 62–74, 80–92, 118–133 (I to IV, respectively) and over the three β-sheets, comprising residues 28–32, 37–44 and 48–56 (A to C, respectively). Both *E. faecalis *and *S. pyogenes *are *Firmicutes *members, and thus are phylogenetically closely related. Therefore, the SufU proteins from the two microorganisms are expected to be very similar.

### SufU molecular dynamics data analysis

**Figure 3:**
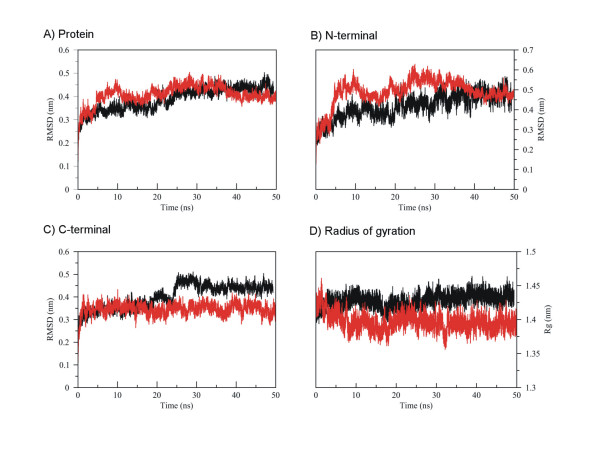
**Root Mean Square Deviation (RMSD) analysis**. Root Mean Square Deviation (RMSD) for the entire protein (A), N-terminal (B) and C-terminal (C) regions, and (D) radius of gyration (template SufU is presented in black and *E. faecalis *SufU in red).

In order to monitor the progress of structural conformational changes in both SufU MD, and check flexibility indices and conformational changes, we evaluated the root mean square deviation (RMSD) of the simulated proteins. Considering the entire protein, both *S. pyogenes *and *E. faecalis *SufU molecules presented similar behaviors, achieving a plateau around 0.45 nm (Fig. 3A). Such increase in protein flexibility appears to be related to the N-(residues 1 to 38) and C-terminal (residues 95 to 136) portions, which may achieve a 0.6 nm deviation from initial conformations (Fig. 3B and 3C). While the degree of conformational modification in *S. pyogenes *SufU points to a role of the crystal environment in protein stabilization, it also indicates a different pattern of folding and/or flexibility around an insertion of 19 amino acids present only in Gram-positive bacteria, responsible for a ~0.1 nm increase in C-terminal RMSD for *E. faecalis *after 20 ns, approximately (Fig. 3C).

The influence of crystal environment on SufU conformation may be observed in the protein secondary structure content (Fig. 2). While the crystallographic structure shows an additional α-helix between helixes III and IV, including residues 105–120, such structural element is not retained on MD simulations, unfolding together with the *S. pyogenes *GPR region (Fig. 2). On the other hand, the *E. faecalis *SufU do not only stabilize this helix, between residues 105 and 120, but also retain a GPR conformation close to that observed in *S. pyogenes *crystal structure. In fact, a series of crystal contacts is observed in such structure, interactions capable to retain the α-helical conformation in this sequence. Being such region conserved between the two proteins, it becomes evident the relevance of the whole protein three-dimensional organization in order to further stabilize and determine the conformational preference of this region.

**Figure 4:**
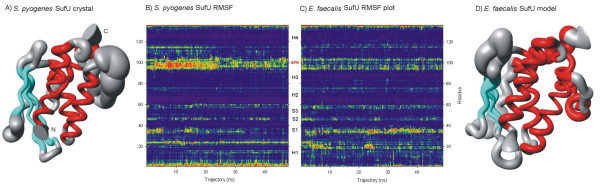
**Flexibility analysis**. Sausage plot for (A) template *S. pyogenes *SufU and (D) model *E. faecalis *SufU proteins. The thickness presented is directly related to the flexibility in the area. Loops are presented in gray, α-helices in red and β-strands in blue. Root Mean Square Fluctuation (RMSF) analysis, as a function of both residue number and time for (B) template *S. pyogenes *and (C) *E. faecalis *SufU proteins, presenting the four α-helices (H), the three β-strands (S), and the GPR region.

While the data presented in RMSD plots gives a global perspective of the protein conformational modifications over the MD trajectory, it lacks resolution at a residue level. So, in order to gain further insights into the conformational behavior of the simulated proteins, we employed a strategy that describes the structural fluctuation of a protein structure as a function of both time and residue number [36-38]. Such root mean square fluctuation (RMSF) analysis confirmed the role of both N-terminal and GPR in SufU flexibility (Fig. 4B and 4C). These fluctuations may be observed in a sausage plot (Fig. 4A and 4D).

*E. faecalis *SufU showed alterations in the GPR, but in a lesser degree than in *S. pyogenes *SufU. Areas related to the active sites of SufU showed higher flexibility too, especially the ones related to residues Cys^35^, Asp^37 ^and Cys^60^, and areas between residues 20 to 25, and 40 to 45. Although the amino acid residues in these parts aren't involved in the active site for [Fe-S] cluster formation, these areas circumvent those residues, once flexibility required for cluster formation is related to the active site of the protein, and modifications established in these regions are essential for enzyme activity.

### Comparison of Gram-negative IscU and Gram-positive SufU scaffold proteins

The structural differences between IscU/SufU proteins from Gram-positive and Gram-negative bacteria are not limited to the GPR region. Also, the N-terminal of such molecules presents differences in both size and sequence (Fig. 1), which is consequently related to the conformation of this sequence. In fact, the analysis of IscU and SufU structures from several organisms (presented in PDB), such as from *Mus musculus *(PDB:1WFZ), from *H. influenzae *(PDB:1R9P), and from *Bacillus subtilis *(PDB:2AZH) support the observation that the N-terminal portion of IscU (verified in both *M. musculus *and *H. influenzae*), is mostly unfolded in comparison to an α-helix in the N-terminal part of both Gram-positive *B. subtilis *SufU (resolved by NMR) and *S. pyogenes *SufU (resolved by crystallography). However, the structural basis for such difference in folding between the two classes of bacteria is not reported. So, we performed an *H. influenzae *IscU molecular modeling experiment using the crystallographic structure of *S. pyogenes *SufU as the template, further refined through MD simulations.

The MD of *H. influenzae *model, retaining a Gram-positive folding demonstrated an unstable conformation, with a progressive unfolding of both N- and C-terminal regions. Such conformational modification was also observed in the radius of gyration, indicating that the Gram-positive folding pattern is not stable in a Gram-negative sequence. Most important, it indicates that these two bacteria proteins indeed present distinct folding in accordance with the topology verified in NMR for IscU.

According to RMSF plots, proteins high flexibility similarities were observed in areas comprising mainly the active sites, the area circumventing them and regions which correspond to the GPR and LPPVK in Gram-negative bacteria.

As observed in the phylogenetic analysis, *Firmicutes *SufU protein and its orthologues in *Proteobacteria *(*Azotobacter vinelandii*), *Gamaproteobacteria *(*H. influenzae*) and *Eucaryota *(*M. musculus*) were clustered in well-defined separated groups. Unlike in the SUF system, the ISC system assembles several other factors which interact directly with the IscU scaffold protein. For example, Hsc66 (HscA) and Hsc20 (HscB) correspond to a specialised chaperone system that selectively binds the [Fe-S] cluster template protein IscU, in which HscA interacts with the LPPVK (residues 99–103) conserved signature [39], stimulating the rate of cluster transfer through an ATP-dependent process by more than 20-fold [40]. SufU does not contain this LPPVK region, which is in accordance with the absence of HscA and HscB genes from the Gram-positive operon.

As discussed above, the SufU crystal and molecular dynamic trajectory structural analysis enabled the identification of a region between the second and third residue of the conserved cysteines. This region is located between helices III and IV, with a loop pattern in the *S. pyogenes *SufU, indicating the possible helix-turn-helix motif. The same region is found in several Gram-positive microorganisms, always related to the scaffold protein for [Fe-S] clusters formation, and presenting the pattern of 19 amino acid residues, with the conserved signature xxFSxxxQGxExxxxLG. This region is apparently susceptible to induced folding upon interaction with target proteins, which MD is capable of refining. Since the conserved residues of this region do not include all of the nineteen residues, and as there are several differences between the non-conserved residues, which could stabilise the helix, the differences in the secondary structure are understandable; nevertheless, conserved residues showed similar flexibility in both SufU RMSF plots.

Considering the structural similarity of both SufU and IscU proteins, molecular modeling of IscU, with posterior dynamics trajectories, enabled us to demonstrate high fluctuation data over the region near the conservative signature LPPVK, which is topologically situated in the same area as the 7 conservative residues in the region of nineteen residues that is only present in Gram-positive bacteria SufU. The GPR region presents itself inside proteins related to scaffold proteins for the [Fe-S] cluster. In addition, the fact that it is a helix-turn-helix domain located between helices III and IV categorises it as a possible protein interaction region. Furthermore, it is possible that GPR is present in SufU: there may be a new factor, not yet characterised, that is present in the *Firmicutes *phylum that could interact with the SufBCD complex and stimulate [Fe-S] cluster assembly and/or transfer to target proteins, given that ATPase activity has already been demonstrated in SufC and has already been characterised for HscA/HscB and IscU of Gram negative bacteria. This hypothesis is reasonable since GPR is located in the same position of the LPPVK conserved signature. To elucidate this further, we are carrying out more experiments to analyse the interaction between SufU and the SufU-mutant and GPR with SufS and/or SufBCD, and the capacity of the complex to assembly and/or release the [Fe-S] cluster cofactor.

## Conclusion

The biological formation of iron-sulfur [FeS] clusters involves the presence of specific biosynthetic machineries and subsequent insertion into specific target proteins. Several [Fe-S] protein machineries have been described in bacteria, in which each (namely NIF, ISC or SUF) presenting specific characteristics. Until now, most of these studies have been performed with *E. coli *and other model bacteria and few studies have considered the [Fe-S] cluster machinery from *Firmicutes *phyla and the Gram-positive bacteria. As well, a small number of studies had been dedicated to the structural and conformational characterization of [Fe-S] cluster machinery individual elements in relation to its specific functions.

So, in order to contribute to fulfill this lack of information the current work demonstrate that SUF appears to be the only machinery involved in [Fe-S] cluster assembly and/or repair system both in enterococci and in other related genera inside the Gram-positive bacteria, whereas includes one ISC element (IscU orthologue) named SufU. The SUF orthologous proteins include SufC, SufD, SufS and SufB. Other genes from *E. coli *SUF machinery, such as SufR, SufE and SufA, were not observed in enterococci. Considering the importance of scaffold proteins in the [Fe-S] cluster biosynthetic machinery, the SufU component was further characterized by means of comparative modeling and molecular dynamics simulations in order to obtain further insights concerning the relation between its structure and conformation and biological function, employing as SufU model protein for *S. pyogenes *and for *E. faecalis *elements. Such analysis had pointed a high flexibility in residues in the active site and mainly adjacent structural elements (residues 20–40, 53–55, 58–60), as well as in a turn between residues 95–115, which is conserved in this type of protein and in *Firmicutes*.

The [Fe-S] cluster formation strategy raises a number of intriguing and still unanswered questions, especially regarding the mechanism of Fe and S mobilisation and assembly within the protein machineries, and also the possible specialisation of these machineries in terms of the type of cluster they can produce, and the control of the target protein transfer step, which probably involves protein-protein interactions. Further studies involving MD data would be attractive in order to both visualise possible structural alterations and to attempt to copy, *in silico*, the biochemical data of the desulfuration process and sulfur delivery from the SufS to the SufU active site, through docking experiments and molecular dynamic refinement, taking into consideration the SufU high flexibility regions here described. In addition, biochemical data aimed at cloning, expression and purification of key SUF machinery proteins will corroborate descriptions of the [Fe-S] cluster biosynthetic pathway in *E. faecalis*, and elucidate this process in several other Gram-positive bacteria, as well as provide insights on its use as a possible molecular target for development of selective therapeutic strategies.

## Methods

The *E. faecalis V583 *genome sequence used in this study is available at the Genbank website .

### Sequence homology search

Searches for protein orthologues in different genomes were performed using the genomic BLAST (tblastn) program at the National Center for Biotechnology Information (NCBI) website  and the DOE JOINT Genome Institute . EBI-EMBL ClustalW  allowed multiple alignment and analysis of consensus sequences and conserved residues (all proteins coded in the three main machineries of [Fe-S] cluster biosynthesis were screened and the conserved residues were analysed according to their primary sequence). All simulations were performed using the GROMACS simulation suite and GROMOS96 force field [41,42]. The Swiss-PDB Viewer [43], DSSP [44], and PROCHECK programs were used in protein analyses, while PyMOL [45] was used for molecule visualisation.

### Molecular Modeling

Comparative modeling using SwissModel was carried out on *E. faecalis *genomic sequence employing *S. pyogenes *SufU structure as template (PDB code 1SU0). Further refinement of *E. faecalis *model was performed by means of MD simulations (see further) [33].

### MD simulations

Each protein, in its monomeric states, was solvated in a dodecahedron box using periodic boundary conditions and the SPC water model [46]. Counter ions were added to neutralise the systems. The MD protocol was based on previous MD studies [38]. Briefly, each system was submitted to energy minimization using the Steepest Descents algorithm. Temperature and pressure were kept constant by coupling protein, ions, and solvent to external temperature and pressure baths, with coupling constants of τ = 0.1 and 0.5 ps, respectively. The dielectric constant was treated as ε = 1, and the reference temperature was adjusted to 310 K. The systems were slowly heated from 50 to 310 K, in steps of 5 ps, each one increasing the reference temperature by 50 K. The total time of SufU simulation was 50 ns.

## Abbreviations

MD: molecular dynamics; GPR: gram positive region.

## Authors' contributions

GPR carried out the primary structure, molecular modeling and molecular dynamics simulations and analysis. HV participated in the design of the study and the bioinformatics analysis. JF conceived the study and participated in its design and coordination and helped draft the manuscript. All authors read and approved the final manuscript.

## Supplementary Material

Additional file 1 **[Fe-S] cluster scaffold IscU/SufU alignments.***Firmicutes *SufU and *Proteobacteria *IscU protein sequences alignment. Click here for file

Additional file 2 **[Fe-S] cluster scaffold IscU/SufU phylogenetic analysis.** Phylogenetic analyses of IscU and SufU protein sequences forming well-defined clades. Click here for file
