# Supplementary material for: Structural studies of the Enterococcus faecalis SufU [Fe-S] cluster protein
Source: BMC Biochem. 2009 Feb 2;10:3. doi: 10.1186/1471-2091-10-3 (PMC2644719; doi:10.1186/1471-2091-10-3)
Supplement: Additional file 1 — [Fe-S] cluster scaffold IscU/SufU alignments.Firmicutes SufU and Proteobacteria IscU protein sequences alignment. [file 1471-2091-10-3-S1.pdf]

```
                        10        20        30        40        50        60        70        80        90       100
                    ....|....|....|....|....|....|....|....|....|....|....|....|....|....|....|....|....|....|....|....|....|
Enterococcus        MA-----LSK---------LDNLYRQVILDHSSHPHH-HGTLDA-SSQTIELNN--PTCGDVIELDVAIE-DGV-IKDIAFQGSGCSISTASASMMTDAV
Carnobacterium      MA-----LTK---------LDQLYRQVILDHSSHPHH-HGKLEQ-STTQIELLN--PTCGDVLQLQLVVK-DQV-IKDIRFDGSGCTISQASASMMTDAV
Lactococcus         MA-----LSK---------LDNLYRAVILDHSSNPRH-AGELQT-G-CMVDLNN--PTCGDVIRLTVEFE-NDV-ISNIAFSGHGCTISTASASMMTVAV
Streptococcus       MA-----LSK---------LNHLYMAVVADHSKRPHH-HGQLDG-V-EAVQLNN--PTCGDVISLTVKFD-EDK-IEDIAFAGNGCTISTASSSMMTDAV
Lactobacillus       MG-----LSK---------LNGLYREVILDYADHPHN-KGELAT-TTNAMTLHN--PTCGDTINLQVEVE-DNK-IKNIAYTGDGCTISQASASMMTDAV
Leuconostoc         MS-----LHN---------LDLLYRQTVMEYAQHPHHYKPMLGT-ETYHVRKYN--PTCGDIIDLAFEMT-DDK-VTDIYFYGDGCAISKASASMMTDLV
Lysinibacillus      MS-----FNN---------LDQLYRSVIMDHYKNPRN-KGSLEG-EAVTIDMNN--PTCGDRIHLTLKVT-DGV-VEDAKFEGEGCSISMSSASMMTQLI
Geobacillus         MS-----SNHP--------LDQLYRQVIMDHYKNPRN-RGVLEG-TNVDVNMNN--PTCGDRIHLTMKVE-DGK-VVDVKFEGEGCSISMSSASMMTQAI
Bacillus            MS-----FNN---------LDQLYRQVIMDHYKNPRN-KGSLED-GSLTVDMNN--PTCGDRIHLHMKVE-DGK-VTDAKFDGEGCSISMASASMMTQIV
Exiguobacterium     MD-----FNN---------LDHLYRQVIMDHYKTPRN-RGAIDG-G-VTIDMNN--PTCGDTIRLQLAIE-DDI-VKDAKFDGEGCSISMASASMMTQAV
Staphylococcus      MN-----FNN---------LDQLYRSVIMDHYKNPRN-KGVLDN-GSMTVDMNN--PTCGDRIRLTFDIE-DGI-IKDAKFEGEGCSISMASASMMTQAV
Paenibacillus       MQ-----LDD------------LYRRVIMDHYKNPRN-RGTLNT-DAVTINLNN--PTCGDRISLQLQVE-DGK-VIDAKFSGEGCSISLSSASMMTEAV
Symbiobacterium     MN-----LSA------------LYQQVILDHYKKPRN-KGAVER-AVLKKHLHN--PTCGDDIEVQVSLGEDGR-IDDVKWNGRGCSISMASASMMSVAL
Acholeplasma        MD-----LKT------------LYRSVIMDHYKNPKN-KGLINDDSYLTVHLNN--PTCGDDLIVQLLIK-DSK-IIDLKQQGKGCSICCASASVASELL
Oenococcus          MG-----LEG---------LNQLYRQVILDAAVDQRY-RGQLLN-PTAVQLAHN--PNCGDVLELQIKVS-DRH-ITAIAFQGQGCTISQASASILADLA
Pediococcus         MSN----IQ------------ELYQKIIVNHAKYPIG-VGVLPQ-YNYHAKLKN--PDCGDDIEVYLKVV-DQH-LEQISFTGQGCIISQASASMMVDAL
Peptostreptococcus  MD-----IR------------EIYTEIITEESRNTKN-KRYLEH-PTHEEKGHN--PSCGDEITLQLDIE-DGI-IKDASYVGVGCAISQASTSLMIDLI
Finegoldia          ME-----LG------------SLYTELILEKSRDKSN-RRELEH-PTHCELGHN--PSCGDEITLQLKVQ-DDT-IEDIAYTGMGCAISQASTSIMCDVI
Clostridium         MD-----LN------------AIYTELIMEHSTSKHN-KRNLDN-PDIKEKGHN--PSCGDEITLELKLN-NGV-IEDLAFTGQGCAISQASTSIMIDLI
Thermoanaerobacter  MSD----LN------------QLYSEVIMEHYENSPH-RRELKD-ATHKERGHN--PLCGDDITLYLKMN-GDI-IEDASFTGHGCAISQASTSMMIDLI
Coprococcus         MQN---NIQ----------NRNFYNEILTEHNMRPEF-KYDLPA-ANVVLEGVN--PNCGDDIWLKLKVE-NGV-IEDGSFVGDGCAISQASADIMLGMI
Nitrococcus         MSD----------------LRDLYQEVILDHNKHPRN-FRSVEP-HSHQADGYN--PLCGDRVTIQLSVEDG--MIASIGFQGDGCAISTASASIMTEVL
Nitrosococcus       MSE----------------LLDLYQEVILDHRKRPRN-FHSMEN-ADFQADGHN--PLCGDRVTVFLKMHNG--IIEDVSFQGEGCAISIASASLMTESL
Polaromonas         MSD----------------LRDLYQELIVDHYRHPHN-FGPLAG-ANRRAEGFN--PLCGDRLTLYLQVVDG--VIENARFEGSGCAISTASASLMTDAL
Coxiella            MSD----------------LNDLYHQLIIDHGRNPRH-FGRLEM-PTHTHEGYN--PLCGDRLTVFFQEKNG--VITDARFEGSGCAISMASASLMMEAL
Myxococcus          MSASD------------D-LKDLYQEVVLEHSKRPRN-YRVVEG-ATAEAAGHN--PLCGDQLVVTLKVEGG--VIKDVAFQGQGCAISKASASLMTGAV
Stigmatella         MSS--------------E-LKDLYQEVVLDHGKRPRN-FRAVEG-ANHRAEGFN--PLCGDQLSVALKVEDG--VIRDIGFQGQGCAISRASASLMTGAV
Anaeromyxobacter    MSE----------------LTDLYQEVVLDHGKRPRN-YGPLEG-ATHRAEGLN--PLCGDRITVAARLEGG--VVRDVRFEGSGCAISKASASVMTGVA
Thauera             MSGMQ------------DSLRELYQEVIFDHNRNPRN-YRLLPA-ANHHADGHN--PLCGDQLTVYLQVEDG--IVRDASFVGHGCAISTASASLMTEAV
Rickettsiella       MMS---------------HLRELYQEMILDHGRNPRH-HHTMPL-ANRLAHGFN--QICGDRLTVYLKIDHG--KIKAISFQGSGCAIAIASASLMSEIL
Methylococcus       ----------------------------MED--------------ANRTVEGFN--PLCGDRITLYVKIDDAG-VIRDVSFQGSGCAISTASASLMTEIV
Legionella          MSM---------------ELRELYQEIIIDHNRNPRN-HHAMED-ATTEAKGFN--PLCGDKLTVYLKLQGD--LIRDVSFIGCGCAISQASASLMTDAL
Dichelobacter       MND----------------LSDLYQELILDHSKNPRN-FHALTP-CTHSATGHN--PLCGDNLKVFVRLNEG--VIADLSFVGDGCAISKASASMMTELA
Mariprofundus       MFE----------------LRDLYQQVIVDHNKSPRN-FGKLAS-FNHEADGYN--PLCGDKLHIYLNVSDDG-IIEDVSFEGEGCAISVASASLMTDAL
Psychromonas        MND---------------ELRALYQEVIIDHGRRPRN-SKKLEH-PSCTQEGYN--PLCGDRLTLYLRIDDN--RIIDASFEGEGCAISMAASSLMTERI
Nitrosospira        MTT-----------------KSLYQEVILDHNRKPRN-YGKLDK-ASHHAVGHN--PLCGDHLDIDLNVEGK--HIEGIAFHGESCAICKASASMMTTVV
Rhodoferax          MSADP---------------KALYQEVILDHNRKPRN-YGELDH-PSHHAEGVN--PLCGDHIHVALDLKGD--SVERIAFHGESCAICKASASMMTVAV
Nitrosomonas        MSL-----------------KSIYQEVILDHNRKPRN-YGALRS-PTHHATGHN--PLCGDRIELDINMLDG--HIEEIAFQGESCAICKASSSMMTNAV
Acidovorax          MTGFTGNA-----------ENELYQEVVLEHKRAPRN-FGHLPQ-PTHQAQGTN--PSCGDRIAVELQLQQG--RVQDIRFTGQGCAICMASTSMMTEAV
Burkholderia        MNPAASHTVPDDA------QSTLYQELVVEHKRAPRH-FGRLAE-PTHEARGHN--PQCGDDLKVQLRIEGG--RIGDIRFDGHGCAICIASASMMTEAV
Acidiphilium        MSGAAD-------------VGDLYQRLIMERARAPLH-AGRPAR-FDAEAEGDN--PMCGDRVHLRLSC-AGG-AIGEVWHETRGCAICVASADLMADAV
Gluconacetobacter   MD-----------------QDGLYQRQVIERARAPVH-AGPLDG-ATHQGEGTN--PMCGDRVRLGVTLDAAG-RVVMVRHQTRGCAICVASADMMADLA
Thermosinus         ----------------------MYTEKVMDHFTNPRN-VGEIEDAN---GIGEVGNAKCGDIMRIYLKIE-NDI-IKDVKFKTFGCGAAIATSSMVTEMV
Desulfitobacterium  ----------------------MYTEKVMDHFMNPRN-VGEIEDAN---GVGEVGNAKCGDIMRIYLDVE-GDI-IKDVKFKTFGCGAAVATSSMVTEMV
Heliobacterium      ----------------------MYTDKVMDHFMNPRN-VGEIENAS---GVGEVGNASCGDIMRIYLDVE-DNI-IKDVKFKTFGCGAAIATSSMVTELI
Desulfotomaculum    ----------------------MYTEKVMDHFTNPRN-VGEIENAD---GIGQVGNPSCGDIMKITLKVE-DNI-IKDIKFKTFGCGAAVATSSMVTEMA
Moorella            ----------------------MYSEKVMDHFTNPRN-VGEIENAD---GVGQVGNPVCGDIMRLYIKVE-DGI-IKDVKFKTFGCGAAIATSSMVTEMV
Carboxydothermus    ---------------------MMYSEKVMDHFTNPRN-VGEIPDAD---GVGEVGNPSCGDIMRIYIKVD-GDK-ITDVKFKTFGCGAAIATSSMVTEMV
Halothermothrix     ----------------------MYSEKVMEHFNNPRN-VGEIKDAD---AVGEVGNPVCGDIIKLYLKIK-DDT-IEDIKFKTFGCGAAVATSSMVTELV
Pelotomaculum       ----------------------MYSEKVIEHFSNPRN-VGEIPDAD---GVGEIGNQVCGDITKIFIKVE-DNV-IKDIKFKTFGCGAAIASGSMLTEMA
Natranaerobius      ----------------------MYNEKVMDHFQNPRN-VGEVESPD---GVGEVGNVTCGDIMRISIKVN-NNEEIEDIKFKTFGCGAAIATSSIVTEMA
Anaerofustis        ----------------------MYNDIVLDHFQNPRN-VGVIEDAT---VVAKEASPSCGDTTEFFLKID-DNDVITDIKFRTFGCAAAIASASMSTELI
Desulfuromonas      ----------------------MYTDKVMDHFSNPRN-VGQIENPN---VVVKVGDPGCGDAVLIFLKID-DN-VITDVKYKVYGCGAAIATTSMASTMV
Pelobacter          ------------------MSFAHYSPKVFDHFLTPRN-NGVLEDAN---GIGEIGDPECGDHLKVYVKIE-DD-IVKDIKFQIKGCPAAIACASAMTELV
Geobacter           -------------------MAEIYSAKVWDHVRNPRN-VGSLEDAN---VVVQAGDPTCGDAVLYFLRIE-ED-IVRDIKFLIKGCGAAIATSSVATELV
Desulfococcus       ---------------------MIYSKTVMDHFRNPRN-VGVIENAA---GVGEVGNPICGDMMTIYLDIQ-DD-RIADIKFQTFGCGSAIAVSSMLTELA
Anaerotruncus       ---------------------MLYSDKVLEHFANPHN-VGEIENAD---GIGEVGNAKCGDIMKMYLKI--KDGVIEDAKFKTFGCGAAIATSSMATDMI
Faecalibacterium    -------------------MASMYSAKVMEHFANPHN-VGELPDAN---GVGEVGNPKCGDIMRMYLKI--ENNVIVDVKFLTFGCGAAIATSSMATDLI
Anaerostipes        ----------------------MYSEKVMDHFEHPRN-VGEIEGAS---GVGTVGNAKCGDIMRIYFDID-DNQIIQDVKFKTFGCGAAVATSSMATELV
```

```
                         10        20        30        40        50        60        70        80        90        100
                     ....|....|....|....|....|....|....|....|....|....|....|....|....|....|....|....|....|....|....|....|....|
Dorea                ----------------------MYSAKVMDHFEHPRN-VGEIEDAS---GVGTVGNAKCGDIMRIYLDID-ENQIIKDVKFKTFGCGAAVATSSMATEMV
Ruminococcus         ----------------------MYTEKVMDHFQHPRN-VGELENAS---GVGTVGNAKCGDIMRIYLDID-ENQIIRDVKFKTFGCGAAVATSSMATELV
Alkaliphilus         ----------------------MYSEKVMDHFMNPRN-VGEIEKAQ---AVGEVGNAKCGDIMKMYLQI--ENDVIVDVKFKTFGCGSAIATSSMATEMI
Ehrlichia            ---------------------MSYSESLLEHYKNPKN-VGTLPKEDYNVGTGLVGAPSCGDVMKLQIKVD-DNGKIIDAKFKTFGCGAAIAASSLATELI
Cowdria              ---------------------MSYSESLLEHYKNPKN-VGTLPKEDYNVGTGLVGAPSCGDVMKLQIKVD-DNGKIIDAKFKTFGCGAAIAASSLATELI
Anaplasma            ---------------------MSYSDAVLDRCKNPQN-VGSLPKDDLNVGTGLVGAPSCGDVMKLQIKVD-ENGTIVDAKFKTFGCGAAIAASSLATERI
Wolbachia            ---------------------MSYNEKILDHYENPRN-VGSLDKNDPNVGTGLVGAPSCGDVMKLQIKVN-DKGVIEDAKFKTFGCGSAIASSSLLTEMI
Plesiocystis         ---------------------MAYSDKVIDHYENPRN-VGSLDKSDEAVGTGIVGAPACGDVMKLQIKV--EDDVITEAKFKTYGCGSAIASSSLVTEWV
Sorangium            ---------------------MAYSDKVIEHYENPHN-VGTLDKNDERVGTGLVGAPACGDVMRLQIKVG-EGGVIEDAKFKTFGCGSAIASSSLATEWL
Oceanospirillum      ---------------------MAYSDKVIDHYENPRN-VGKMNDKDSNVGTGMVGAPACGDVMRLQIKVS-DEGIIEDAKFKTYGCGSAIASSSLVTEWV
marine_ proteobac.   ---------------------MAYSEKVIDHYENPRN-VGKLDDSSKNVGTGMVGAPACGDVMRLQIQVS-SDGIIEDAKFKTYGCGSAIASSSLLTEWV
Pseudomonas          ---------------------MAYSEKVIDHYENPRN-VGKLDAADPNVGTGMVGAPACGDVMRLQIKVN-EQGVIEDAKFKTYGCGSAIASSSLATEWM
Azotobacter          ---------------------MAYSDKVIDHYENPRN-VGKLDAQDPDVGTGMVGAPACGDVMRLQIKVN-EQGIIEDAKFKTYGCGSAIASSSLATEWM
Colwellia            ---------------------MAYSEKVLDHYENPRN-VGSMDKNDPSVATGMVGAPACGDVMKLQLKIS-DDGIIEDAKFKTYGCGSAIASSSLVTEWV
Moritella            ---------------------MAYSEKVIDHYENPRN-VGSFDKNDPSIATGMVGAPACGDVMKLQLKIN-DDGIIEDAKFKTYGCGSAIASSSLVTEWV
Shewanella           ---------------------MAYSEKVLDHYENPRN-VGSFDKNDPSVVTGMVGAPACGDVMKLQLRID-ADGVIEDAKFKTYGCGSAIASSSLVTEWV
Aeromonas            ---------------------MAYSEKVIDHYENPRN-VGGFDKNDPSIATGMVGAPACGDVMKLQLKIS-DDGIIEDAKFKTYGCGSAIASSSLVTEWV
Pseudoalteromonas    ---------------------MAYSDKVIDHVENPRN-VGVLDKNDPSVATGMVGAPACGDVMKLQIKVS-ADGIIEDAKFKTYGCGSAIASSSLVTEWV
Alteromonadales      ---------------------MAYSDKVIDHVENPRN-VGALDKNDPSVATGMVGAPACGDVMKLQIKVS-DTGVIEDAKFKTYGCGSAIASSSLVTEWV
alpha_proteobac.     ---------------------MAYSEKVIDHYENPRN-VGGFDKDDKNIATGMVGAPACGDVMKLQLKVG-ENGVIEDAKFKTYGCGSAIASSSLVTEWV
Haemophilus          ---------MAFTRIEKRKVKMAYSEKVIDHYENPRN-VGSLDKKDSNVGTGMVGAPACGDVMQLQIKVD-DNGIIEDAKFKTYGCGSAIASSSLITEWV
Pasteurella          ---------------------MAYSNKVIDHYENPRN-VGSLDKKDANVGTGMVGAPACGDVMQLQIKVS-EEGIIEDAKFKTYGCGSAIASSSLITEWV
Actinobacillus       ---------------------MAYSEKVIDHYENPRN-VGTFDKEAADIGTGMVGAPACGDILRLQIKVN-DQGIIEDARFKAYGCGSAIASSSLITEWV
Mannheimia           ---------------------MAYSEKVIDHYENPRN-VGTFDKEASDTGTGMVGAPACGDILRLQIKVN-DQGIIEDARFKAYGCGSAIASSSLITEWV
Neisseria            ---------------------MAYSDKVIDHYENPRN-VGTFDKNDESVGTGMVGAPACGDVMRLQIKVN-DEGIIEDAKFKTYGCGSAIASSSLITEWV
Vibrio_cholerae      ---------------------MAYSEKVIDHYENPRN-VGSFDKEDPSVGSGMVGAPACGDVMRLQIKVS-PEGIIEDAKFKTYGCGSAIASSSLVTEWV
Vibrionales          ---------------------MAYSEKVIDHYENPRN-VGSFDKEDPSVGSGMVGAPACGDVMKLQIKVT-PEGIIEDAKFKTYGCGSAIASSSLVTEWV
Photobacterium       ---------------------MAYSEKVIDHYENPRN-VGSFDKDDQNVGSGMVGAPACGDVMKLQIKVT-EEGIIEDAKFKTYGCGSAIASSSLITEWV
Shigella             ---------------------MAYSEKVIDHYENPRN-VGSFDNNDENVGSGMVGAPACGDVMKLQIKVN-DEGIIEDARFKTYGCGSAIASSSLVTEWV
Escherichia_coli     ---------------------MAYSEKVIDHYENPRN-VGSFDNNDENVGSGMVGAPACGDVMKLQIKVN-DEGIIEDARFKTYGCGSAIASSSLVTEWV
Salmonella           ---------------------MAYSEKVIDHYENPRN-VGSFDNNDDNVGSGMVGAPACGDVMKLQIKVN-DEGIIEDARFKTYGCGSAIASSSLVTEWV
Enterobacter         ---------------------MAYSEKVIDHYENPRN-VGSFDNSDESVGSGMVGAPACGDVMKLQIKVN-NEGIIEDARFKTYGCGSAIASSSLVTEWV
Klebsiella           ---------------------MAYSEKVIDHYENPRN-VGSFDNSDENVGSGMVGAPACGDVMKLQIKVN-NEGIIEDARFKTYGCGSAIASSSLVTEWV
Photorhabdus         ---------------------MAYSEKVIDHYENPRN-VGSFDSEDPMVGSGMVGAPACGDVMKLQIKVN-NEGIIEDARFKTYGCGSAIASSSLVTEWM
Xenorhabdus          ---------------------MAYSDKVIDHYENPRN-VGSFDSEDPSVGSGMVGAPACGDVMRLQIKVN-NEGIIEDARFKTYGCGSAIASSSLVTEWM
Serratia             ---------------------MAYSEKVIDHYENPRN-VGSFDNEDPSVGSGMVGAPACGDVMKLQIKVN-NEGIIEDARFKTYGCGSAIASSSLVTEWM
Yersinia             ---------------------MAYSEKVIDHYENPRN-VGSFDSQDPTIGSGMVGAPACGDVMKLQIKVN-EAGIIEDARFKTYGCGSAIASSSLVTEWM
Erwinia              ---------------------MAYSEKVIDHYENPRN-VGSFDNADPSIGSGMVGAPACGDVMKLQIKVN-NEGIIEDARFKTYGCGSAIASSSLVTEWV
Providencia          ---------------------MAYSEKVIDHYENPRN-VGSFDNNDPSVGSGMVGAPACGDVMKLQIKVN-DNGIIEDARFKTYGCGSAIASSSLVTEWM
Buchnera             ---------------------MAYSKKVMDHYENPRN-VGSFSNSDSNVGSGLVGAPACGDVMKLQIKVN-EQGIIEDACFKTYGCGSAIASSSLVTEWI
Janthinobacterium    ---------------------MAYSDKVLDHYENPRN-VGAFDKGDETIGTGMVGAPACGDVMKLQIKVG-ADGVIQDAKFKTYGCGSAIASSSLVTEWV
Herminiimonas        ---------------------MAYSDKVLDHYENPRN-VGAFDKGDETIGTGMVGAPACGDVMKLQIKVG-ADGVIEDAKFKTYGCGSAIASSSLVTEWV
Azoarcus             ---------------------MAYSEKVLDHYENPRN-VGAFGKEDEGVGTGMVGAPACGDVMKLQIKVG-KDGVIEDAKFKTYGCGSAIASSSLVTEWV
Thiobacillus         ---------------------MSYSDKVLDHYENPRN-VGAFNKEDEGVGTGMVGAPACGDVMKLQIKVG-KDGLIEDAKFKTYGCGSAIASSSLVTEWV
Ralstonia            ---------------------MSYSNKVLDHYENPRN-VGSFDKGDGTVGTGMVGAPACGDVMKLQIKVN-EQGVIEDAKFKTYGCGSAIASSSLVTEWV
Dechloromonas        ---------------------MSYSVKVIDHYENPRN-VGSFAKEDDGVGTGMVGAPACGDVMKLQIKVN-KSGVIEDAKFKTYGCGSAIASSSLVTEWV
Chromobacterium      ---------------------MAYSEKVLDHYENPRN-VGSFDKGDDSIGTGMVGAPACGDVMKLQIKVG-ADGVIEDAKFKTYGCGSAIASSSLVTEWV
Limnobacter          ---------------------MAYSEKVLDHYENPRN-VGSFEKGDDEVGTGMVGAPACGDVMKLQIKVG-EDGVITDAKFKTYGCGSAIASSSLVTEWV
Bordetella           ---------------------MAYSSKVLDHYENPRN-VGSFDKGDESVGTGMVGAPACGDVMKLQIKVS-ESGVIEDARFKTYGCGSAIASSSLVTEWV
Polynucleobacter     ---------------------MAYSEKVIDHYENPRN-VGSFEKGDDSVGTGMVGAPACGDVMKLQIRVN-DQGVIEDAKFKTYGCGSAIASSSLVTEWV
Methylibium          ---------------------MAYSEKVVEHYENPRN-VGSFEKGDDTVGTGMVGAPACGDVMKLQIKVNPATGLIEDAKFKTYGCGSAIASSSLVTEWV
Leptothrix           ---------------------MAYSDKVIDHYEHPRN-VGAFEKGDESVGTGMVGAPACGDVMKLQIKVDPATGKIADARFKTYGCGSAIASSSLVTEWV
Delftia              ---------------------MAYSDKVVDHYENPRN-VGSFDKNDESVGTGMVGAPACGDVMKLQIKVNPETGVIEDARFKTYGCGSAIASSSLVTEWV
Comamonas            ---------------------MAYSEKVVDHYENPRN-VGSFDKSDDSVGTGMVGAPACGDVMKLQIKVNPATGVIEDARFKTYGCGSAIASSSLVTEWV
Verminephrobacter    ---------------------MAYSEKVVDHYENPRN-VGSFDKGDDSVGTGMVGAPACGDVMKLQIKVNPQTGVIEDARFKTYGCGSAIASSSLVTEWV
Methylobacillus      ---------------------MAYSDKVLDHYENPRN-VGSLDKNDPSVGTGMVGAPACGDVMKLQIKVN-DQGIIEDAKFKTYGCGSAIASSSLVTELI
Methylophilales      ---------------------MAYSDKVLDHYENPRN-VGSFDKNDPSVGTGMVGAPACGDVMKLQIKIS-DNGMIEDAKFKTYGCGSAIASSSLVTEWL
Acinetobacter        ---------------------MAYSEKVIDHYENPRN-VGVLDKNSENVGTGMVGAPACGDVMRLQIQVN-DNGVIEEARFKTYGCGSAIASSSLVTEWL
```

```
                             10        20        30        40        50        60        70        80        90        100
                    ....|....|....|....|....|....|....|....|....|....|....|....|....|....|....|....|....|....|....|....|....|
Psychrobacter       --------------------------MAYSNQVIDHYENPRN-VGNLDKDAKNVGTGMVGAPACGDVMRLQIQVG-DDGIIEDARFKTYGCGSAIASSSLVTEWL
Candidatus          --------------------------MAYGEKVMDHYENPRN-VGVLDKDAKNVGTGMVGAPACGDVMRLQIQVD-DNGVIEDAKFKTYGCGSAIASSSLLTEWV
Endoriftia          --------------------------MAYSDKVLDHYENPRN-VGAFDNEEKSVGTGMVGAPACGDVMRLQIKVN-DAGVIEDARFKTYGCGSAIASSSLLTEWV
Magnetococcus       --------------------------MAYNEKVLDHYEKPRN-VGSMDKDADDVGTGMVGAPSCGDVMKLQIKVD-DNGVIEDAKFKTFGCGSAIASSSLVTEWI
Magnetospirillum    --------------------------MAYSDKVIDHYEHPRN-VGALDKDDSAVGTGLVGAPACGDVMKLQIKVS-AEGIIEDAKFKTFGCGSAIASSSLVTEWV
Rickettsia          ------------------------MTMAYSKKVIDHYENPRN-VGSLDKENKNVGTGLVGAPACGDVMKLQIAVD-DDGIITDAKFKTFGCGSAIASSSLVTEWV
Neorickettsia       --------------------------MAYSDSVLNHYNNPRN-VGTIDKNKKNVGTGLVGAPACGDVMRLQIEVD-NNGCIVDAKFKTFGCGSAIASSSLATEYL
```

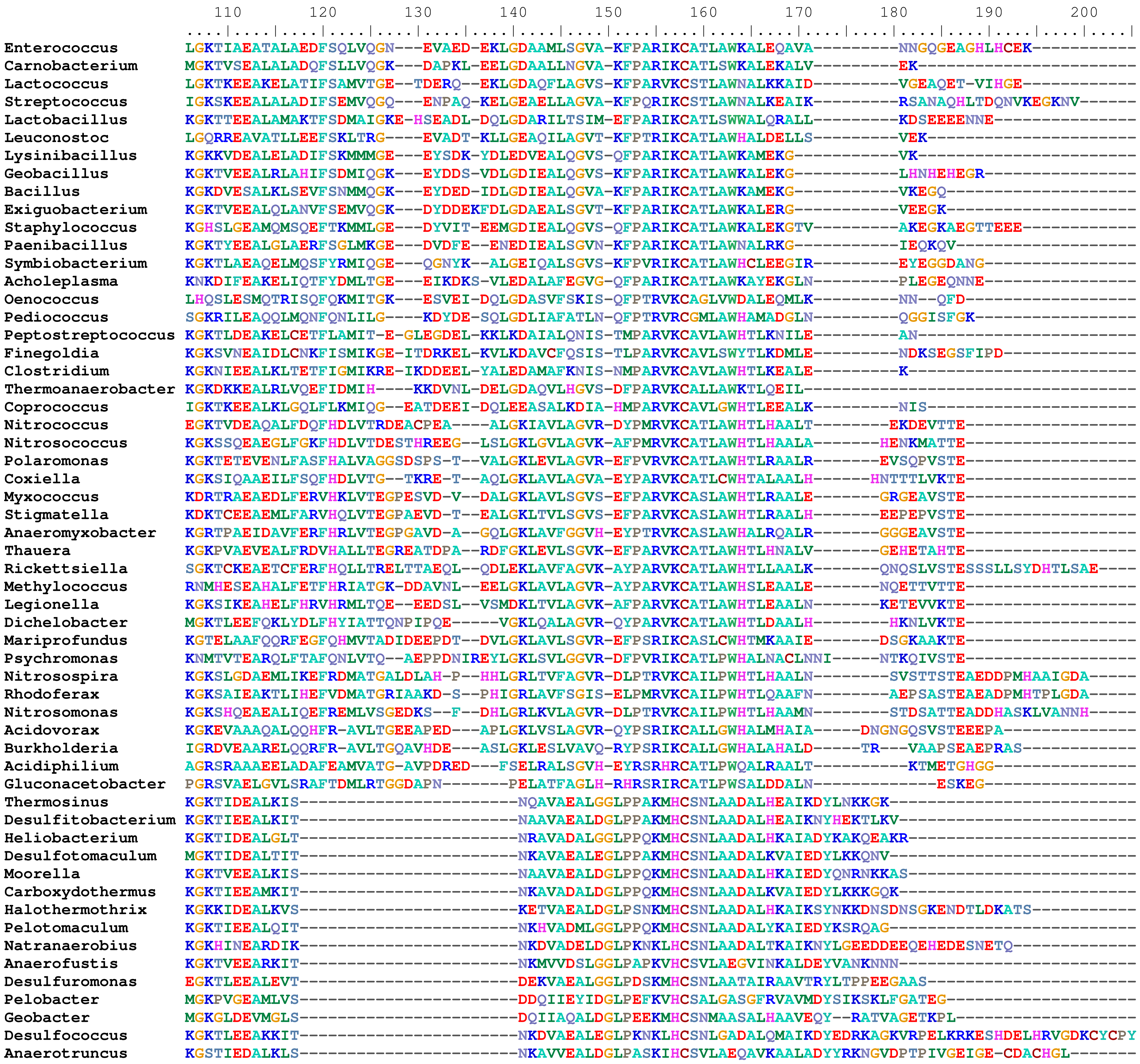

110
120
130
140
150
160
170
180
190
200
Enterococcus
Carnobacterium
Lactococcus
Streptococcus
Lactobacillus
Leuconostoc
Lysinibacillus
Geobacillus
Bacillus
Exiguobacterium
Staphylococcus
Paenibacillus
Symbiobacterium
Acholeplasma
Oenococcus
Pediococcus
Peptostreptococcus
Finegoldia
Clostridium
Thermoanaerobacter
Coprococcus
Nitrococcus
Nitrosococcus
Polaromonas
Coxiella
Myxococcus
Stigmatella
Anaeromyxobacter
Thauera
Rickettsiella
Methylococcus
Legionella
Dichelobacter
Mariprofundus
Psychromonas
Nitrosospira
Rhodoferax
Nitrosomonas
Acidovorax
Burkholderia
Acidiphilium
Gluconacetobacter
Thermosinus
Desulfitobacterium
Heliobacterium
Desulfotomaculum
Moorella
Carboxydothermus
Halothermothrix
Pelotomaculum
Natranaerobius
Anaerofustis
Desulfuromonas
Pelobacter
Geobacter
Desulfococcus
Anaerotruncus

```
                         110       120       130       140       150       160       170       180       190       200
                      ....|....|....|....|....|....|....|....|....|....|....|....|....|....|....|....|....|....|....|....|
Faecalibacterium      KGKTVDEALKLT-----------------------NKAVVEALEGLPPVKVHCSVLAEQAVKAALSDYYRRQGIDPEPIVGKLEEDCEHCESCGH-----
Anaerostipes          KGKSVQEALEVT-----------------------NKAVMEALDGLPPVKVHCSLLAEEAIHAALWDYAQKNGIKIEGLEKPKSDIGEEEVE--EEY---
Dorea                 KGKTVQEAMEVT-----------------------NKAVMEALDGLPPVKVHCSLLAEEAIHAALWDYAQKNGITIEGLQKPKSDIHEGEEDEAEEY---
Ruminococcus          KGKNIQEAMKVT-----------------------NKAVMEALDGLPPVKVHCSLLAEEAIHAALWDYAEKNGIKIEGLEKPKTDIHEDEEE--EEY---
Alkaliphilus          KGKTVKEALQLT-----------------------NKAVAEALDGLPPVKMHCSVLAEQAVKAAIYNYAKENNVHYEELEGFVPDEDHDHHDHEEEE---
Ehrlichia             KGKTIDEAHQLK-----------------------NTVLAKELS-LPPVKIHCSLLAEDAVKAAIKDYNMKQATKKDKLSSSNE----------------
Cowdria               KGKTVDEAHELK-----------------------NTVLAKELS-LPPVKIHCSLLAEDAVKAAINDYHMKQANKKNATKDPNE----------------
Anaplasma             KGKTIEEACMLK-----------------------NTVLAKELS-LPPVKIHCSLLAEDAVKAAVHDYKSKQQIAKGDKC--------------------
Wolbachia             KGRTISDVTQIK-----------------------NTQIVEELS-LPPVKIHCSVLAEDAIKAAIHDYQSKQKN--------------------------
Plesiocystis          KGMKIDEAEQIK-----------------------NSQIAEELS-LPPVKIHCSVLAEDAIKAAIADYRDKKKG--------------------------
Sorangium             KGKTIDEAETIK-----------------------NSMIAEELH-LPPVKIHCSVLAEDAIKSAIADFRAKQQAKRELAGAAPSVPAAAAATASAE----
Oceanospirillum       KGMSLDEAAELK-----------------------NTSIAEELA-LPPVKIHCSVLAEDAIKAAVADYKEKQDK--------------------------
marine_ proteobac.    KGKHIDDAAEIK-----------------------NTEIAEELA-LPPVKIHCSVLAEDAIKAAVRDIREKQGS--------------------------
Pseudomonas           KGKTLDEAETIK-----------------------NTTIAEELA-LPPVKIHCSVLAEDAIKAAVRDYKQKKGLL-------------------------
Azotobacter           KGRTLEEAETIK-----------------------NTQIAEELA-LPPVKIHCSVLAEDAIKAAVRDYKHKKGLV-------------------------
Colwellia             KGKSIEEAGEIK-----------------------NTAIAEELA-LPPVKIHCSILAEDAIKAAIEDYKSKQSK--------------------------
Moritella             KGKSIEQAGEIT-----------------------NMTIAEELE-LPPVKIHCSILAEDAIKAAIDDYKKKKAS--------------------------
Shewanella            KGKTIEEAAAIK-----------------------NTDIAEELA-LPPVKIHCSILAEDAIKAAIDDYKSKQDK--------------------------
Aeromonas             KGKTLDEAAGIK-----------------------NTDIAEELA-LPPVKIHCSILAEDAIKAAIADYKQKKGL--------------------------
Pseudoalteromonas     KGKTLEQAATIK-----------------------NTDISAELE-LPPVKIHCSILAEDAIQAAIANYKSKQAK--------------------------
Alteromonadales       KGKTLDEAATIK-----------------------NTDISAELE-LPPVKIHCSILAEDAIQAAIADYKSKQAK--------------------------
alpha_proteobac.      KGKTLDEATTIK-----------------------NTDIAEELA-LPPVKIHCSILAEDAIKAAVADYKAKNA---------------------------
Haemophilus           KGKSLEEAGAIK-----------------------NSQIAEELE-LPPVKVHCSILAEDAIKAAIADYKAKQG---------------------------
Pasteurella           KGKSLDEAQAIK-----------------------NSQIAEELE-LPPVKVHCSILAEDAIKAAIADYKAKKEAK-------------------------
Actinobacillus        KGKSLEEAGAIK-----------------------NSDIAEELE-LPPVKVHCSILAEDAIKAAIADYKEKQAK--------------------------
Mannheimia            KGKSLEEAGAIK-----------------------NSDIAEELE-LPPVKVHCSILAEDAIKAAIADYKEKQAK--------------------------
Neisseria             KGKSLDDALAIK-----------------------NSEIAEELE-LPPVKIHCSILAEDAVKAAVADYRKRQENR-------------------------
Vibrio_cholerae       KGKSIDEAAAIK-----------------------NSEIAEELE-LPPVKIHCSILAEDAIKAAVADYKKKHQH--------------------------
Vibrionales           KGKSIDEAAAIK-----------------------NSEIAEELE-LPPVKVHCSILAEDAIKAAVADYKKKR----------------------------
Photobacterium        KGKTLDEAASIK-----------------------NSAIAEELE-LPPVKVHCSILAEDAIKAAVSDYKKKHEQK-------------------------
Shigella              KGKSLVEAQAIK-----------------------NTDIAEELE-LPPVKIHCSILAEDAIKAAIADYKSKREAK-------------------------
Escherichia_coli      KGKSLDEAQAIK-----------------------NTDIAEELE-LPPVKIHCSILAEDAIKAAIADYKSKREAK-------------------------
Salmonella            KGKSLDEAQAIK-----------------------NTDIADELE-LPPVKIHCSILAEDAIKAAIADYKSKREAK-------------------------
Enterobacter          KGKSLDEAQAIK-----------------------NTDIADELE-LPPVKIHCSILAEDAIKAAIADYKSKREAK-------------------------
Klebsiella            KGKSLDEAQAIK-----------------------NTDIADELE-LPPVKIHCSILAEDAIKAAIADYKSKREAK-------------------------
Photorhabdus          KGKSLEQAEAIK-----------------------NTEIADELE-LPPVKIHCSILAEDAIKAAIADYKSKRQGR-------------------------
Xenorhabdus           KGKSLEQAEAIK-----------------------NTQIADELE-LPPVKIHCSILAEDAIKAAIADYKSKRQAK-------------------------
Serratia              KGKSLDQAEAIK-----------------------NTQIAEELE-LPPVKIHCSILAEDAIKAAIADYKSKHSAK-------------------------
Yersinia              KGKSLEQAEAIK-----------------------NTQIAEELE-LPPVKIHCSILAEDAIKAAIADYKSKHTAK-------------------------
Erwinia               KGKSLNEAEAIK-----------------------NTQIAEELE-LPPVKIHCSILAEDAIKAAIADYKSKRDAQ-------------------------
Providencia           KGKSLDEAEAIK-----------------------NTAIAEELE-LPPVKIHCSILAEDAIKAAIADYKSKRQGK-------------------------
Buchnera              KGKSITEAEAIK-----------------------NTSIVEELE-LPPVKIHCSILAEDAIKAAISDYKSKKNKN-------------------------
Janthinobacterium     KGKTLDQAMSIK-----------------------NTQIAEELA-LPPVKIHCSILAEDAIKAAVLDYKTKHGVAESKEAA-------------------
Herminiimonas         KGKTLDQALSIK-----------------------NTQIAEELA-LPPVKIHCSILAEDAIKAAVLDYKTKHGVAEAEKAV-------------------
Azoarcus              KGKTVDQALEIK-----------------------NTQIAEELA-LPPVKIHCSILAEDAIKAAVADYKKKQG---------------------------
Thiobacillus          KGKTLDQAMEIK-----------------------NTAIAEELA-LPPVKIHCSILAEDAIKAAVADYKQKKGLE-------------------------
Ralstonia             KGKTLDQALEIR-----------------------NTQIAEELA-LPPVKIHCSILAEDAIKAAVADYKEKHGTAEQKAA--------------------
Dechloromonas         KGKTVDQALSIK-----------------------NTEIAEELA-LPPVKIHCSILAEDAIKAAVADYKKKHGE--------------------------
Chromobacterium       KGKSLDEALAIK-----------------------NTAIAEELA-LPPVKIHCSILAEDAIKAAVEDYKQKHGK--------------------------
Limnobacter           KGKTLDQALEIK-----------------------NTAIAEELA-LPPVKIHCSILAEDAIKAAVEDYKKKHA---------------------------
Bordetella            KGKTLDEALDIR-----------------------NTQIAEELA-LPPVKIHCSILAEDAIKAAVKDYKDKHAAPADAQGETAAAAVAN-----------
Polynucleobacter      KGKTLDQALEIK-----------------------NSLIAEELA-LPPVKIHCSILAEDAIKAAVADYKEKHPAQ-------------------------
Methylibium           KGKSLDEALTIK-----------------------NTQIAEELA-LPPVKIHCSILAEDAIKAAVSDYKAKHGNAQPAAATATH----------------
Leptothrix            KGKTLDEALTIK-----------------------NTHIAEELA-LPPVKIHCSILAEDAIKAAVDDYKAKH--AQ-------------------------
Delftia               KGKTLDEAAALK-----------------------NSQIAEELA-LPPVKVHCSILAEDAIKAAVDDYRAKRTAVVAA----------------------
Comamonas             KGKTLDEAAALK-----------------------NSQIAEELA-LPPVKVHCSILAEDAIKAAVNDYRAKRTATEA-----------------------
Verminephrobacter     KGKTLDEAAAVK-----------------------NSEIAQELA-LPPVKIHCSILAEDAIKAAVLDYKTKRMAAAAVA---------------------
Methylobacillus       KGKSLDQAMEIK-----------------------NSDIAQELA-LPPVKIHCSVLAEDAIRAAVADLKAKQGAKDQQVA--------------------
Methylophilales       KGKTLDQASEIK-----------------------NSAIAEELA-LPPVKIHCSVLAEDAIKAAVADLKSKQ--K-------------------------
```

```
                      110       120       130       140       150       160       170       180       190       200
                  ....|....|....|....|....|....|....|....|....|....|....|....|....|....|....|....|....|....|....|....|
Acinetobacter     KGKTLDEAQAIK-----------------------NIDIATELA-LPPVKVHCSVLAEDAIKAAIEDYRSKKSKA-------------------------
Psychrobacter     KGKTLDQASEIK-----------------------NKHIAEELA-LPPVKVHCSVLAEDAIKAAIEDYRAKSGTVEA-----------------------
Candidatus        KGKTLNEAAEIK-----------------------NSDIAEELS-LPPVKIHCSVLAEDAIKAAINDIKSKA----------------------------
Endoriftia        KGKTLDEAQQIK-----------------------NSEIAEELA-LPPVKVHCSVLAEDAIKAAIKDYTDKNAG--------------------------
Magnetococcus     KGKTIDEALTIK-----------------------NKDVAEELA-LPPVKVHCSVLAEDAIKAAVKDYREKQQKKGA-----------------------
Magnetospirillum  KGKTLDEAASIK-----------------------NTDIAQELA-LPPVKIHCSVLAEDAIKAAIADYKKKSG---------------------------
Rickettsia        KGRSVEDAETIK-----------------------NTEIAKELS-LPPVKLHCSLLAEDAIKAAIADYKQKKESKKDS----------------------
Neorickettsia     IGKSIEEAEKIK-----------------------NTEIASTLC-LPPIKMHCSMLAEDAIKAAIKDFREKQVTSSTEEAGNENTENKS-----------
```
