# Supplementary figures and images for: Structural studies of the Enterococcus faecalis SufU [Fe-S] cluster protein
Source: BMC Biochem. 2009 Feb 2;10:3. doi: 10.1186/1471-2091-10-3 (PMC2644719; doi:10.1186/1471-2091-10-3)

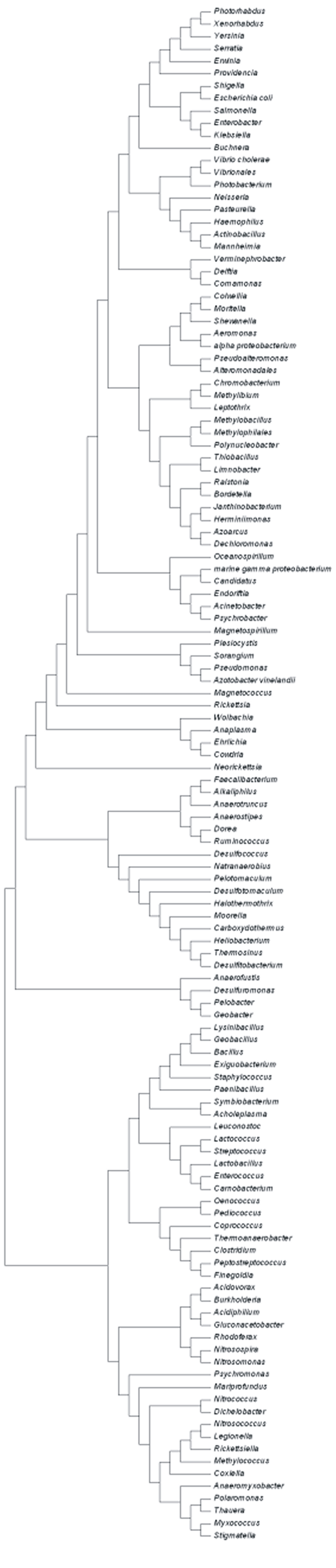

Supplement: Additional file 2 — [Fe-S] cluster scaffold IscU/SufU phylogenetic analysis. Phylogenetic analyses of IscU and SufU protein sequences forming well-defined clades. [file 1471-2091-10-3-S2.png]
